# Dissolution and biodurability: Important parameters needed for risk assessment of nanomaterials

**DOI:** 10.1186/s12989-015-0088-2

**Published:** 2015-04-28

**Authors:** Wells Utembe, Kariska Potgieter, Aleksandr Byron Stefaniak, Mary Gulumian

**Affiliations:** National Institute for Occupational Health, PO Box 4788, Johannesburg, 2000 South Africa; University of Malawi, Malawi Polytechnic, Blantyre, Malawi; National Institute for Occupational Safety and Health, Morgantown, USA; University of Witwatersrand, Johannesburg, South Africa

**Keywords:** Micro-sized and nano-sized particles, Solubility, Dissolution, Biodurability, Biopersistence, Kinetics

## Abstract

Biopersistence and biodurability have the potential to influence the long-term toxicity and hence pathogenicity of particles that deposit in the body. Therefore, biopersistence and biodurability are considered to be important parameters needed for the risk assessment of particles and fibres. Dissolution, as a measure of biodurability, is dependent on the chemical and physical properties (size, surface area, etc.) of particles and fibres and also of the suspension medium including its ionic strength, pH, and temperature. *In vitro* dissolution tests can provide useful insights as to how particles and fibres may react in biological environments; particles and fibres that release ions at a higher rate when suspended *in vitro* in a specific simulated biological fluid will be expected to do so when they exist in a similar biological environment *in vivo*. Dissolution of particles and fibres can follow different reaction kinetics. For example, the majority of micro-sized particles and fibres follow zero-order reaction kinetics. In this case, although it is possible to calculate the half-time of a particle or fibre, such calculation will be dependent on the initial concentration of the investigated particle or fibre. Such dependence was eliminated in the shrinking sphere and fibre models where it was possible to estimate the lifetimes of particles and fibres as a measure of their biodurability. The latter models can be adapted for the dissolution studies of nanomaterials. However, the models may apply only to nanomaterials where their dissolution follows zero-order kinetics. The dissolution of most nanomaterials follows first-order kinetics where dependence on their initial concentration of the investigated nanomaterials is not required and therefore it is possible to estimate their half-times as a measure of their biodurability. In dissolution kinetics for micro-sized and nano-sized particles and fibres, knowledge of dissolution rate constants is necessary to understand biodurability. Unfortunately, many studies on dissolution of nanoparticles and nanofibres do not determine the dissolution rates and dissolution rate constants. The recommendation is that these parameters should be considered as part of the important descriptors of particle and fibre physicochemical properties, which in turn, will enable the determination of their biodurability.

## Introduction

Biopersistence of mineral particles and fibres is defined as the extent to which they are able to resist chemical, physical, and other physiological clearance mechanisms in the body [1]. Biopersistence is considered to be one of the main contributors to mineral particle and fibre toxicity and hence pathogenicity. Biodurability, defined as the ability to resist chemical/biochemical alteration, is a significant contributor to biopersistence. For example, the lower pathogenicity of micro-sized chrysotile fibres compared to tremolite fibres has been attributed to its lower biodurability and biopersistence [2].

Dissolution, defined as release of molecules and/or ions, of particles and fibres has been used as a measure of their biodurability [1,3,4]. The determination of dissolution rates has therefore provided an insight on how a certain particle or fibre may interact with its biological and environmental surrounding. If particles or fibres release ions at a fast rate, their short-term toxic effect could be identical to those of the dissolved ions [5]. On the other hand, if the particles release ions at a slow rate, there is a greater likelihood that the particles will be the cause of the observed adverse effects [6].

There is sizeable literature on biopersistence, biodurability and dissolution of natural and synthetic micro-sized particles and fibres. The past few decades have seen the rapid development of engineered nanomaterials such as nanoparticles and nanofibres with dimensions in the nanoscale (1 – 100 nm). With increasing and widespread use of these nanomaterials, their health and environmental effects have become of concern. It has been proposed that the concepts applied for dissolution evaluations of micro-sized particles and fibres can also be usefully applied to engineered nanoparticles and fibres [7]. It was therefore suggested that the study of the biodurability and biopersistence of engineered nanomaterials in biological systems and the assessment of their dissolution rates are important parameters to be determined for their risk assessment [8]. For example, for some nanoparticles such as silver, there have been discussions as to whether the nanoparticles themselves could be toxic due to their size and shape or due to the release of silver ions [9]. It has also been shown that dissolution plays a substantial role in cytotoxicity of some nanoparticles such as ZnO, where the ions are toxic, compared to CeO_2_ where the toxicity is attributed to the nanoparticle itself [10]. Hence, understanding nanomaterial characteristics and associated biodurability will provide insights for assigning nanomaterials to a mode of action category and guide decision making for a tiered toxicity testing strategy (involving *in vitro* and *in vivo* tests) for risk assessment. As such, the risk assessment for nanomaterials with low biodurability may only require short- term toxicity tests while those with very long biodurabilities may require long-term chronic toxicity testing. This will have implications in the cost of the risk assessment and has already been suggested for micro-sized particles and fibres [1]. In turn, such data could be used to develop biological- or health-based occupational exposure limits for nanomaterials.

This paper presents a narrative literature review on dissolution and biodurability of micro-sized particles and fibres *in vitro* as well as a review on dissolution of nanomaterials with the aim of assessing the applicability of the concepts used in the study of the biodurability and dissolution of micro-sized particles and fibres to engineered nanoparticles and nanofibres.

### Methodologies for assessing biodurability

The dissolution rate of particles and fibres, as a measure of their biodurability, may be determined using *in vivo* (short and long term) and *in vitro* (*acellular* or *cellular*) tests [1,11]. *In vivo* studies have been used for assessing the biopersistence of nanoparticles through the assessment of their clearance and biodurability. These *in vivo* tests are based on intratracheal instillation or inhalation exposures, two methods accepted by the European Commission [12]. The clearance of the particles and fibres is then measured after 3 months by sacrificing the test animals and lavaging the macrophages for analysis [13]. The biodurability on the hand other hand, is assessed through the decrease of the particle or fibre diameter. *Cellular in vitro* systems for assessing biodurability involve treatment of cells in culture with particles or fibres followed by the examination of the intracellular particles and fibres using microscopy to determine the change in their diameter [14-17]. For *cellular* dissolution tests, alveolar macrophages are commonly used as a suitable test system for particles and fibres [17,18]. A number of limitations exist with *cellular* systems, including the fact that cells will not be in their normal natural environment [17] and that the volumes of media used are small compared to *in vivo* systems [1].

*Acellular in vitro* testing involves the determination of the degree of dissolution of micro-sized particles and fibres as well as nanoparticles and nanofibres using simulated biological and environmental fluids. *Acellular in vitro* tests are often used to screen for dissolution as they provide information on how particles and fibres may behave in a true biological or environmental system. For example, in a series of studies with the same beryllium oxide particles (200 nm diameter), dissolution rate estimates in an *acellular in vitro* model [19], *cellular in vitro* model [20], and dog *in vivo* model [21] agreed within a factor of two, demonstrating the utility of *in vitro* tests to predict *in vivo* systems. In a study of micro-sized uranium oxides, there was reasonably good agreement between dissolution measured using an *acellular in vitro* model and a rat *in vivo* model [22]. There are numerous simulated biological or environmental fluids that can be used for dissolution studies. Some of the most used simulated fluids include body fluids which represents human blood plasma, simulated interstitial fluid representing fluids deep in the lung, simulated macrophage lysosomal fluid, and simulated gastric and intestinal fluids representing the digestive system. Simulated environmental fluids include among others sea water and river water [23].

The dissolution rates in *acellular in vitro* systems are assessed by the changes in the sample mass of the particles and fibres, concentration of the ions released into the simulated fluid or a physicochemical characteristic for the particles or fibres [24]. As such, *acellular in vitro* systems tend to be more amenable for well-characterized nanomaterials and less is known about their utility for more complex matrices such as nanomaterials embedded in polymer materials. The physicochemical characteristics of the particles and fibres, for example, can include the decrease in their diameter over time [25-29]. The dissolution rate in turn, may be determined by a linear plot of the change in mass, concentration or particle/fibre characteristic against time [28,30-33]. Although *cellular in vitro* and animal *in vivo* systems may provide more biologically plausible environments, *acellular in vitro* systems are easy to implement and provide a rapid and cost effective first hand alternative to the other two testing systems [22,34-37]. As such, these *acellular* test systems are the focus of the remainder of this review article.

### Acellular *in vitro* dissolution tests for particles and fibres

The dissolution of particles and fibres in the lungs can occur intracellularly and extracellularly [1]. Therefore, *in vitro acellular* tests for studying the dissolution rates in the lungs involve use of a neutral fluid such as Gamble’s fluid (pH of 7.2 – 7.8), which represents the interstitial lung fluid and airway lining fluid [38]. Particles and fibres may be phagocytosed by the pulmonary alveolar macrophages and therefore, dissolution rates are also investigated in a fluid known as artificial lysosomal fluid (ALF, pH 4.5 – 5) to simulate the environment within vesicles in the macrophages [13,38-40].

*Acellular* dissolution studies are conducted using either static or dynamic methods. The static method involves the exposure of known masses of the particles or fibres to a fixed volume of simulated fluid in a beaker. The particles or fibres are either placed in a dialysis membrane, isolated from the fluid using filters, or are freely added to the simulated fluid. When a dialysis membrane is employed the particles are suspended in the simulated fluid inside the dialysis tubing. Over time, a concentration gradient develops between the particle-laden fluid inside the membrane and the particle-free fluid in the beaker and the dissolved ions migrate into the exposure media. To determine the degree of dissolution, the simulated fluid is analysed for the specific ions or molecules of interest [30,32,41-47]. With filters, the particles or fibres are placed in between two filters and sealed along the perimeter; as dissolved ions migrate into the media they are quantified to determine the degree of dissolution. Unlike dialysis membranes, a potential limitation of filters is that the pore size must be sufficiently small enough to prevent migration of nanomaterials into the simulated fluid thereby biasing measurements of dissolved mass. Colloidal particle sizing techniques such as dynamic light scattering can be used to verify the efficacy of the filter barrier. In contrast, when the particles are added directly to the media, the particles are stirred for a period and thereafter ultracentrifugation or filtration is used to separate the particles from the dissolved ions for measurement [28,33,43,48-54]. The use of fractionation or separation techniques can be a source of bias for materials that dissolve rapidly because the particles or fibres will dissolve further during centrifugation, etc. [55,56]. The chemical composition of the fractionation membrane can also be a source of bias. For example, Kennedy et al. reported that the cellulose membrane in a centrifugal filtration device bound silver ions, thereby underestimating dissolution rate [57]. Moreover, static systems face challenges of the occurrence of saturation of the exposure media, since the volume is limited. This leads to the inhibition of dissolution when equilibrium is reached.

In contrast, the continuous-flow-through (CFT) dynamic system involves a flow-through chamber containing the particles or fibres with a membrane that separates them from the flowing fluid. The particles or fibres are suspended in the test media and contained in a separate chamber inside the CFT unit. The test fluid flows over the membrane and any dissolved ions or molecules from the particles or fibres migrate through the membrane into the fluid and are collected using a fraction collector. Limitations of filters also apply to the CFT dynamic method. Currently, the smallest commercially available pore sizes of filter membranes are in the order of tens of nanometers. As such, very small nanomaterials (e.g., quantum dots) or fast dissolving nanomaterials that quickly decrease in size may not be suitable for study with techniques that employ filter barriers to isolate the test material from simulated media. As with the static test, efficacy of the barrier membrane to isolate nanomaterials from the simulated media can be verified using colloidal sizing techniques.

The CFT dynamic method of dissolution testing is therefore thought to be more representative of dissolution occurring in biological surroundings and hence is recommended to avoid reaching an equilibrium which may restrict dissolution [58]. One drawback, though, with the CFT systems is the need for large volumes of test fluid to maintain flow rate [59], and the low reproducibility within laboratories due to different experimental conditions implemented [24].

### Concepts in dissolution of particles and fibres

Most dosing techniques in toxicity assessments require the test material to be in a liquid phase where the terms “in solution” or “solubility” are used. The introduction of particles or fibres to a liquid medium with the intention of making a “solution” will involve dispersion. Thus for particle colloids (particles in the 1 nm - 1 μm size range), the term *dispersed* rather than *dissolved* is used to produce a “dispersion” and not a “solution”; the former term is where the solid material co-exists with a liquid phase [60]. Therefore, dispersion refers to the distribution of the particles or fibres themselves in a volume of liquid. Dissolution, in the case of particles or fibres, denotes the release of ions or molecules (solute) from the surface of a material and their distribution throughout the available liquid volume as a result of entropy. Therefore, the terms “solubility” and “in solution” may not be appropriate in particle dissolution chemistry.

As per above definitions, the dissolution of particles or fibres is different from the solubility of inorganic salts. When considering the solubility of inorganic salts such as sodium chloride (NaCl), this term implies that the NaCl crystals will disintegrate into the compositional ions Na^+^ and Cl^−^. In this case, the solubility of a solute is the analytical composition of a saturated solution, expressed in terms of the proportion of a designated solute in a designated solvent. It may be expressed as a concentration such as molarity, molality, mole fraction or mole ratio [60]. In contrast, the concentration gradient that exists between the surface of the particle and fibres and the bulk solution (simulated fluid or biological fluid) is the driving force of their dissolution. This depends on the size and surface properties of the particles and fibres, and also on the nature of media, where the release of ions occurs at different rates with different orders of kinetics [61].

### General considerations in reaction kinetics

The biodurability of particles and fibres using *acellular* dissolution tests is based on determination of the rate of dissolution of the particles and fibres. In general, different chemical processes have different reaction kinetics where they can follow either zero-, first- or second-order kinetics. The rate of reaction is expressed in a rate equation where the order of the reaction is the power to which the concentration of the reacting species is raised:

1 $$ r= Rate=-\frac{dM}{dt}=k{\left[ reactant\right]}^n $$

The rate law above describes the change in the concentration of a substance *M* in units of mass or moles per volume, over a period of time *t*, with a rate constant *k* and with the order of reaction *n* being 0, 1, or 2. Although rare, the order of reaction can also be 3, a fractional or even a negative value. The order of a reaction can only be determined experimentally and not from the balanced chemical equation. Also, the rate of any reaction may not be deduced from the order of reaction as a zero-, first-, or second-order reaction may take several seconds to several years to complete [62].

#### Zero-order reactions

When the dissolution follows zero-order kinetics, the rate of reaction will be independent of the concentration(s) of the reactant(s) and therefore the rate law will be expressed as follows:

2 $$ Rate=-\frac{dM}{dt} = k{M}^0 $$

where *M*^*0*^ is the concentration of the reactant, *t* is the time of the reaction and the rate of reaction is equal to the rate constant, *k.* Depending on the units of concentration, *k* will have units of mole per volume per time or mass per volume per time (such as (mol/dm^3^)/s or (g/dm^3^)/s). An example of a zero-order chemical reaction is the oxidation of tetrachloroplatinate (II) (PtCl_4_^2−^) in excess of persulphate ion (S_2_O_8_^2−^) in acidic conditions (HCl) where the reaction kinetics was found to be independent of the concentration of S_2_O_8_^2−^ (5 – 50 mM) [63].

#### First-order reactions

First-order kinetics in contrast, depend on the concentration of at least one reactant and the rate law is given as:

3 $$ -\frac{dM}{dt}=kM $$

where *M* is the concentration of the reactant, and *k* is the rate constant in units of per time (s^−1^, hr^−1^, day^−1^). Therefore, with first-order dissolution processes, the rate of reaction is proportional to the concentration of the reactant, where doubling of the concentration will double the rate [62,64]. However, a reaction can be first-order with respect to one reactant but can have an overall order of greater than 1. Examples of chemical reactions with first order kinetics may include the reaction of ozone with non-ionised solutes, such as aliphatic alcohols, olefins, benzene etc. [65].

#### Second-order reactions

The rate law for a reaction in which the order of dissolution with respect to one reactant is a second-order reaction is given as follows:

4 $$ -\frac{dM}{dt}=k{M}^2 $$

where *M* is the concentration of the reactant raised to the power two with a rate constant *k* in units of per concentration per time such as (L/mol)/s. An example of a chemical reaction with second order kinetics includes the transesterification reaction of soybean oil using methanol [66].

Equations 2, 3 and 4 are integrated to give linear equations from which rate constants can be obtained.

### Dissolution kinetics of natural and synthetic micro-sized particles and fibres and nanomaterials

Extensive research has been devoted to the understanding of the dissolution kinetics of natural and synthetic particles and fibres [25,67-70]. These studies involve the determination of the dissolution rate, rate constant and the order of reaction. In these studies it is important to note that particles and fibres having equal masses and the same chemical composition but different diameters may appear to have different dissolution rates. For this reason, dissolution kinetics involving particles and fibres need to take into account differences in surface area. Therefore, the dissolution rate is expressed using the amount released per surface area rather than per mass [71]. As a result, the dissolution of particles and fibres has been described by specific surface-area-normalized rate laws. For instance, the surface-area-normalised zero-order kinetic dissolution rate law is given as:

5 $$ \frac{dM}{dt} = -{k}_{SSA}A $$

where $\left(\frac{dM}{dt}\right)$ is the rate of the dissolved mass *M* expressed in mass/time such as g/s, which is proportional to the specific surface area (*A*) in contact with the fluid expressed in m^2^/g and *k*_*SSA*_ is the surface-area-normalised zero-order dissolution rate constant expressed in units mass/time.surface area such as g/(s/m^2^) or ng/(hr/cm^2^) [72]. The dissolution constant, *k*_*SSA*_, is a property of the particle composition and does not depend on its size or shape [68].

For fibres with uniform diameter that release ions congruently from an initial mass *M*_*0*_ and initial diameter *D*_*0*_, the dissolution process can be expressed by Equation 6 below [70,72]:

6 $$ 1 - {\left(\frac{M}{M_0}\right)}^{\frac{1}{2}}=\frac{2{k}_{SSA\ }t}{D_0\rho } $$

where *ρ* is the fibre density. These two equations result in an equation that relates (*D(t)*), which is the fibre diameter at time *t* to the initial fibre diameter *D*_*0*_, fibre density *ρ* and the normalized dissolution constant *k*_*SSA*:_

7 $$ D(t)={D}_0-\frac{2{k}_{SSA}\ t}{\rho } $$

where *D* is measured in units of length such as cm and *ρ* in g/cm^3^ [68,73].

For those particles for which the dissolution follows first-order kinetics, the rate law is expressed in terms of not only the specific surface area as is the case with zero-order kinetics but also in terms of the concentration (mass per unit volume) of the particles. The surface area normalised first-order rate law is therefore given as:

8 $$ \frac{dM}{dt} = -{k}_{SSA} AM $$

where the rate of dissolved mass $\left(\frac{dM}{dt}\right)$ in g/s from particles and fibres with concentration *M* is proportional to the specific surface area (*A,* in units of m^2^/g) in contact with the fluid, and *k*_*SSA*_ is the surface-area-normalised first-order dissolution rate constant in units of 1/(m^2^.s)

For second-order reactions, the surface-area-normalised rate law can be expressed as:

9 $$ \frac{dM}{dt} = -{k}_{SSA}A{M}^2 $$

where the rate of dissolution $\left(\frac{dM}{dt}\right)$ in units of concentration (expressed either in molarity or mass concentration) per time of reactant M and *k*_*SSA*_ is the surface-area-normalised second-order dissolution rate constant in units of L/(mol/s) per surface area.

As indicated above, the dissolution rate constant can be obtained by using linear plots of the integrated rate laws. Alternatively, as suggested for nanoparticles, the dissolution rate constants can be determined using a thermodynamic approach in which the dissolution is followed using calculations of Gibbs free energy (G) for the system comprising of nanoparticles, dissolved material and solvent as a function of particle size. This approach has been discussed comprehensively elsewhere [74]. This results in a kinetic equation for the change of the concentration in the solution, denoted as ${c}_L^1(1)$, during the path of the dissolution process.

10 $$ \frac{d{c}_L^1(1)}{dt}=kB\left[{c}_L^1(1)\right]\left|\nabla g\left(r,z\right)\right| $$

The concentration of the particle-forming or dissolving species in the solution ${c}_L^1$ (1) as function of time, *t*, is assumed to be proportional to the magnitude of the gradient of *g*(*r*,*z*). The term *k* is the rate constant, $B\left[{c}_L^1(1)\right]$ is a coefficient, which may depend on the concentration, and ∇ is a mathematical operator that denotes change in gradient. This method has been used by these authors in dissolution studies for number of nanoparticles [75-78]. A drawback with this approach is the difficulty in the determination of the order of reaction. For example, in one of their studies [75] these authors used the rate constant for silica nanoparticles given in units of h^−1^, indicating that the reaction was first-order while in the same study for the same nanoparticles the rate constant was given in units of (mol/m^2^)/s indicating that the reaction was zero-order.

### Factors affecting the dissolution of micro-sized particles and fibres

The dissolution of a material depends on the intrinsic properties of the material such as particle size, composition, shape, crystallinity, and surface modification as well as the extrinsic solvent properties such as pH, ionic strength, constituent solvated molecules, temperature, and concentration [61].

**Figure 1:**
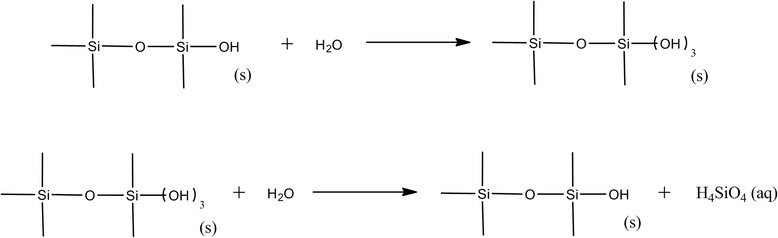
Mechanism of silica particle dissolution. Dissolution of silica particles due to the occurrence of surface saturation at pH 7, adapted from Heaney PJ and Banfield JF [80].

For larger particles and fibres, a material may release ions by different mechanisms depending on the arrangements of the atoms and bonds. For example, silica particle dissolution follows the mechanism shown below in Figure 1 [79].

Figure 1 shows that the dissolution of micro-sized silica particles occurs in a two step process involving saturation of the surface with water resulting in the formation of silicic acid, followed by the severance and breakage of the Si-O bond which releases monomeric silicic acid [79,80]. The breakage of the Si-O bond is the rate limiting step as it requires substantial amount of energy [81], and therefore this step may determine the dissolution rates and the order of reaction [62] which in this case is shown to follow zero-order kinetics [79].

Not only will the bulk composition affect the observed dissolution rates but so too will the crystallinity of particles and fibres. Polymorphs have different crystal structures, and therefore they will have different dissolution patterns. For example, the crystallinity of silica species has been shown to affect the dissolution kinetics since the Si-O bonds, bond length and bond energies differ. It was found that the Si-O bond distance of crystalline mesoporous silica is in the range of 1.598 – 1.625 Ǻ, which is slightly shorter than amorphous silica by approximately 0.02 Ǻ [82]. This suggests that the bond strength of crystalline mesoporous silica should be slightly stronger than that of amorphous silica, and that dissolution in amorphous silica should occur faster. This was found in the study of Icenhower and Dove [79] where the dissolution of amorphous silica was at a faster rate than crystalline silica.

Properties of the simulated fluid also affect the dissolution of particles and fibres and should be tailored to accurately model the biological fluid of interest. For example, the pH of the medium has an effect on the dissolution [83]. For this reason pulmonary dissolution studies are usually conducted at neutral pH as encountered outside cells and at acidic conditions, representing conditions inside the cell (around pH 4.5) and in lysosomes (around pH = 5.5). Ingestion dissolution studies of micro-sized particles, fibres and nanomaterials require the use of simulated gastric and intestinal fluid, representing the gastrointestinal tract. The pH of the *in vivo* gut system varies between different compartments [84], therefore the simulated gastric and intestinal fluids should have different pH values. Simulated gastric fluid typically has a pH of below 2, in contrast to simulated intestinal fluid with a higher pH of around 6.5 [38].

Additionally, the concentration of ions and molecules (sulphides, chlorides, proteins, enzymes, polysaccharides, etc.) in the surrounding media (simulated or biological fluid) can influence dissolution. These molecules and ions have the potential to influence the dissolution rates of particles and fibres by adsorbing onto the surface of the particles [7,85,86]. For example, the presence of chelating agents such as EDTA (ethylenediaminetetraacetic acid) has been shown to significantly increase the release of Ca^2+^ ions from calcite [85]. In another study, chloride ions were reported to have an effect on the dissolution of malachite ore particles where the dissolution rates increased with an increase in chloride concentration [87].

### Factors affecting the dissolution of nanomaterials

Many factors that affect the dissolution of micro-sized particles and fibres will also likely affect the dissolution of nanomaterials. These factors include the media composition, dispersion state of the nanomaterial, and availability of constituents to form complexes with released ions. One important distinction is that nanomaterials have a larger surface-area-to-volume ratio compared to micro-sized particles. This difference causes nanomaterials to have a much greater ability for dissolution compared to micro-sized particles and fibres. In addition, nanoparticles have greater fraction of atoms at the edges and corners instead of planar terraces compared to larger particles. This makes it easier for ions and small clusters at the surface to break away from nanoparticles because of the higher free energy [88]. For nanoparticles there is usually a kinetic size effect, a phenomenon in which an initial high concentration of the dissolved species is observed followed by a decrease in concentration until the saturation is reached [77]. It has been shown that the kinetic size effect increases with decreasing particle size [76,77].

The dissolution rate of nanomaterial is also affected by the presence of strong oxidants such as oxygen, H_2_O_2_, HO_2_^−^ and OCl^−^ and also sunlight and natural organic matter (NOM) in the suspending solution. The oxidising agents will either enhance or suppress the dissolution rate of the nanoparticles. For example, oxygen and H_2_O_2_ will enhance the dissolution rate of silver nanoparticles [52].

Another important factor in the dissolution of nanoparticles is aggregation. Aggregation is defined as secondary particles composed of primary particles that are bonded together (fused, sintered) and acting as a unit [7]. Aggregation occurs when electrolytes (environmental media has a high concentration) present in the media decrease the electrostatic repulsion/barrier between particles [89,90]. Aggregation will decrease available external surface area and hence influence the ion release kinetics of nanoparticles and thus reducing the extent of dissolution [7]. It has therefore been observed that the dissolution rate decreases as the aggregation state of nanoparticles increases [33,91]. Also, when aggregation occurs there will be fewer sites available that can be oxidised [33]. For example, the dissolution of nanorods is reported to be nearly or completely quenched in the aggregated state [92].

To prevent aggregation of nanoparticles, ligands of a stabilizing agent are often chemically bonded to particle surfaces. Ligand-stabilized nanomaterials are generally more stable (remain in a dispersed state) against changes in solution pH and concentration. However, the ligands can affect the dissolution rates of nanoparticles where they can either increase or decrease dissolution [93]. For example, it was found that the dissolution rate for citrate-stabilised nanoparticles was much lower than those observed for PVP (polyvinylpyrrolidone) - stabilised nanoparticles [30]. Therefore, the rate of dissolution will depend on the type of the surface species and the manner in which the agents are attached to nanoparticle surfaces [61].

Similar to larger particles, the simulated biological fluid composition can also cause changes in saturation concentration and dissolution kinetics of nanoparticles [61]. Dissolution of nanoparticles is enhanced when organic or ionic molecules are able to form soluble complexes with the released ions. On the other hand, dissolution is hindered by the formation of less soluble complexes [7]. For example, a small amount of chloride significantly decreased the rate of release of soluble Ag species compared to the chloride-free control [32].

### Biopersistence and biodurability of micro-sized particles and fibres

Removal of fibres and particles from the body (and hence determining their biopersistence) is accomplished by a variety of mechanisms including 1) physical transportation by ciliary clearance, macrophage-mediated and lymphatic clearance, 2) dissolution and 3) breakage and disintegration (for fibres) [4]. The overall clearance process, and hence biopersistence can be expressed by the following kinetic equation:

11 $$ \frac{\ dM}{dt} = -{k}_{\mathrm{overall}}M $$

where $\left(\frac{dM}{dt}\right)$ is the rate of clearance (in units of mass/time) from particles of concentration *M*; *k*_*overall*_ = *k*_*Physi*_ + *k*_*dis*_ + *k*_*decom*_; where *k*_*physi*_ is the physiological clearance comprising of transportation of particle or fibre by physical removal through ciliary, macrophage-mediated, and lymphatic clearance mechanisms; *k*_*dis*_ is the fraction of clearance that is due to dissolution; and *k*_*decomp*_ is particle removal by breakage and disintegration [4]. Hence, the biopersistence of particles and fibres is dependent on their physical clearance as well as on their rate of dissolution as a measure of their biodurability [26]. The above mentioned clearance mechanisms in turn, will depend on the physicochemical properties of particles and fibres such as size, shape, crystallinity, etc. [4,94].

When discussing dissolution-mediated clearance, biodurability can be described by two important parameters: particle (or fibre) dissolution half-time and lifetime. In the following paragraphs we describe equations that are used to calculate the half-times from the dissolution rate constant for zero-, first- and second-order dissolution processes.

For zero-order processes, the half-time of particles or fibres can be calculated using the integrated form of Equation 2 resulting with the equation below:

12 $$ {\mathrm{t}}_{1/2} = \frac{{\left[ reactant\right]}_0}{2{k}_{dis}} $$

where *[reactant]*_*o*_ is the initial concentration of the particles or fibres, *k*_*dis*_ is the dissolution rate constant and *t*_*1/2*_ is the half-time. With this equation, *t*_*1/2*_ may be calculated in seconds, hours or years. For example, if *[reactant]*_*o*_ has units of moles/litre and *k*_*dis*_ has units of moles/litre.seconds, the half-time will have a final unit of seconds. Short dissolution half-times are particularly important for nanomaterials such as ZnO that are thought to exert toxic effects from release of ions [10,95].

Equation 12 also shows that the half-time is directly proportional to the initial concentration of the particles or fibres. Therefore, although it may be possible to apply in the assessment of half-times of particles and fibres, the calculated *t*_*1/2*_ will only be applicable to that specific initial concentration. The half-times of particles and fibres may also be determined using Equation (6) – a plot of $1 - {\left(\frac{M}{M_0}\right)}^{\frac{1}{2}}$ versus time should yield a straight line whose slope (fraction per day) can be determined using non-linear least squares regression to estimate the best fit line for the data. Hence, *t*_*1/2*_ can be calculated as the quotient ln(2)/slope [70].

In addition to half-times, the dissolution lifetimes of particles and fibres with zero-order kinetics may be calculated as a measure of biodurability using the shrinking sphere model (Equation 13) and the shrinking fibre model (Equation 14). These models estimate the lifetimes of particles and fibres from the diameter and molar volume. Note that if the size distribution of the particle or fibre population is monodisperse, the dissolution lifetime will be the same for all constituents. In contrast, if the size distribution of the particle population is polydisperse, the dissolution lifetime is the time required for the largest particle or fibre in the population to completely dissolve. The models are derived from the surface-area-normalised zero-order rate law. The shrinking sphere model is given as:

13 $$ \tau =\frac{d}{2{V}_mk} $$

where τ is the dissolution lifetime (s), *d* is the diameter of the spherical particle (m), *V*_*m*_ is the molar volume (m^3^/mol), and *k* is the rate constant (mol/(m^2^.s) [96].

The dissolution lifetime for the shrinking fibre model is given as [97]:

14 $$ \tau =\frac{3d}{4{V}_mk} $$

The shrinking sphere model has been used to estimate the lifetimes of talc and quartz [96,98]. The shrinking fibre model has been used to estimate the lifetimes of olivine, chrysotile and tremolite asbestos [96,99]. According to Jurinski [96] the approach may be extended to other particles and fibres, provided that the rate constants for their dissolution are available.

Some particles and fibres may have dissolution kinetics that follows rate orders other than zero-order. For particles and fibres that follow first-order dissolution kinetics, the half-times may be calculated from rate constants using the following equation which is derived from the integrated form of Equation 3 [100]:

15 $$ {t}_{1/2}=\frac{ \ln (2)}{{\mathrm{k}}_{\mathrm{dis}}} $$

where *t*_*1/2*_ is the half-time (in units of time) and *k*_*dis*_ is (the non-normalised) dissolution rate constant in 1/units of time.

For particles and fibres that follow second-order dissolution kinetics, the half-times may be calculated using the initial concentration which is derived from the integrated form of Equation 4:

16 $$ {t}_{1/2}=\frac{1}{2{k}_{dis}{\left[ reactant\right]}_0} $$

In this case, the half-time (*t*_*1/2*_) is inversely proportional to the initial concentration of the particles or fibres. Once again, the calculated *t*_*1/2*_ will only be applicable to that specific initial concentration.

The half-times and lifetimes of some particles and fibres which were calculated using the approaches described in this section are given in Table 1.

**Table 1:** **Half-time (calculated from** ***k***_***SSA***_ **) and lifetime (estimated from shrinking sphere/fibre models) of micro-sized particles and fibres**

**Particles/fibres**	**Conditions**	***k***_***SSA***_	**Half-time**	**Lifetime**
WO_3_	As received (aggregated) 36.2 μm particles in artificial airway epithelial lining fluid (pH 7.4)	2.5 ± 0.3 × 10^−5^ g tungsten/(cm^2^·day) [70]	4 ± 1 days^a^	
WO_3_	Dispersed (individual particles) 36.2 μm particles in artificial airway epithelial lining fluid (pH 7.4)	0.9 × 10^−5^ g tungsten/(cm^2^·day) [70]	11 days^a^	
WO_3_	Mixture of 36.2 μm particles and 2.4 μm cobalt particles in artificial airway epithelial lining fluid (pH 7.4)	1.3 ± 0.4 × 10^−6^ g tungsten/(cm^2^·day) [70]	79 ± 23 days^a^	
WO_3_	As received 36.2 μm particles in artificial lung alveolar macrophage phagolysosomal fluid (pH 4.5)	9.8 ± 2.9 × 10^−9^ g tungsten/(cm^2^·day) [70]	9893 ± 2549^a^	
WO_3_	Dispersed (individual particles) 36.2 μm particles in artificial lung alveolar macrophage phagolysosomal fluid (pH 4.5)	4.3 ± 0.4 × 10^−9^ g tungsten/(cm^2^·day) [70]	21541 ± 1890^a^	
WO_3_	Mixture of 36.2 μm particles and 2.4 μm cobalt particles in artificial lung alveolar macrophage phagolysosomal fluid (pH 4.5)	1.1 ± 0.4 × 10^−9^ g tungsten/(cm^2^·day) [70]	8052 ± 2458^a^	
Talc	1 μm particles	1.4 × 10^−11^ mol Si/(m^2^.s) [96]		8 years^b^
Chrysotile	1 μm fibres pH 2 to 6 at 37°C	5.9 × 10^−10^ mol Si/(m^2^.s) [108]		9 months^b^
Olivine	1 μm particles	7.6 × 10^−11^ mol Si/(m^2^.s) [96]		4.8 years^b^
Quartz	1 μm particles	1.4 × 10^−13^ mol Si/(m^2^.s) [96]		5000 years^b^

^a^As determined by Stefaniak [70], ^b^as determined by Jurinski [96].

### Applicability of concepts used in dissolution rate and biodurability studies of micro-sized particles and fibres to nanomaterials

Agreement on all the relevant factors that affect pulmonary clearance of nanomaterials has not been reached [101-103]; therefore, determination of the overall clearance or biopersistence of nanoparticles may not be possible using Equation 11. However, the assessment of biodurability of nanoparticles and fibres may be possible through the assessment of their dissolution rates, half-times, and dissolution rate constants. The same approaches to study biodurability of micro-sized particles and fibres may therefore be applicable to study the biodurability of nanomaterials. As such, the same surface-area-normalised rate laws may apply to the assessment of the biodurability of nanoparticles and nanofibres.

For nanoparticles that may follow zero-order dissolution kinetics, the shrinking sphere model (Equation 13) may apply. Using data from literature we applied this model to synthetic amorphous silica nanoparticles. This exercise utilised the surface-area-normalised rate constants of 7.88 × 10^−13^ mol.m^−2^ s^−1^ for nanoparticles with radius 6.7 nm and 2.57 × 10^−12^ mol.m^−2^ s^−1^ for nanoparticles with radius 3.6 nm as calculated by Roelofs and Vogelsberger [75] and a molar volume of 22.688 cm^3^/mol (22.688 × 10^−6^ m^3^/mol) for bulk silica [104]. This calculation is presented as follows:

$$ \begin{array}{l}{\tau}_{\left(r=6.7\  nm\right)}=\frac{2\left(6.7\times {10}^{-9}\ \right)}{(2)\left(22.688\times {10}^{-6}\right)\left(7.88\times {10}^{-13}\right)}\\ {}\kern3.36em =\frac{1.34\times {10}^{-8}}{3.576\times {10}^{-17}}\\ {}\kern0.96em \tau =3.75\times {10}^8\  seconds=\pm 12\  years\\ {}{\tau}_{\left(r=3.6\  nm\right)}=\frac{2\left(3.6\times {10}^{-9}\right)}{(2)\left(22.688\times {10}^{-6}\right)\left(2.57\times {10}^{-12}\right)}\\ {}\kern3.36em =\frac{7.2\times {10}^{-9}}{1.16\times {10}^{-16}}\\ {}\kern1.08em \tau =6.21\times {10}^7\  seconds= \pm 2\  years\\ {}\kern2.76em \end{array} $$

We have therefore calculated a lifetime of approximately 12 years for 6.7 nm radius and 2 years for the 3.6 nm radius silica nanoparticles. It should be noted that the assumption that nanoparticles may have a similar molar volume as their larger counterparts may not be totally valid. Hence, future studies should consider how to more accurately determine the molar volume for nanoparticles. Admitting that there are different forms of silica, these lifetimes are generally much lower than those estimated for larger silica-based micro-sized particles [96,97]. This is expected since nanoparticles should have higher abilities to release ions than their micro-sized particulate counterparts because of their higher surface-to-volume ratio.

The shrinking sphere model may not be applicable to nanoparticles having first-order dissolution kinetics such as silver nanoparticles [33,52,105], ZnO [106] and TiO_2_ [107]. This is due to the fact that when using the surface-area-normalised first-order rate constant in the shrinking sphere model, the lifetime, τ will have units of mol.s as shown in the equation:

17 $$ \tau\ (mol.s)=\frac{d(m)}{\left[2{V}_m\left(\frac{m^3}{mol}\right)\times k\left(\frac{1}{s.{m}^2}\right)\right]} $$

Instead, the first-order kinetic half-time equation (Equation 15) may be used. Using this equation, and the *k*_*dis*_ (from literature), we could calculate the half-times, as an estimate for their biodurability, of different nanoparticles as presented in Table 2.

**Table 2:** **Dissolution half-times of nanoparticles calculated from** ***k***_***dis***_

**Nanoparticles**	**Conditions**	**Rate constants obtained from literature**	**Calculated half-time**
Ag 4.8 nm	Citrate stabilized	4.1 day^−1^ [52]	0.169 days (4 hours)
Ag 60 nm	Citrate stabilized	0.74 day^−1^ [52]	0.963 days (22.5 hours)
Ag 4.8 nm	Citrate stabilized, in deionised water, at 0.05 mg/L total silver	0.88 day^−1^ [52]	0.79 days (18.9 hours)
Ag 4.8 nm	Citrate stabilized in deionised water, at 0.2 mg/L total silver	0.53 day^−1^ [33]	1.3 days (31.4 hours)
Ag 4.8 nm	Citrate stabilized in deionised water, at 2 mg/L total silver	0.023 day^−1^ [33]	30 days (723 hours)
Ag 10.6 nm	Tris-HOAc buffer in 10 M H_2_O_2_ 0.001 nM Ag^o^	0.128 day^−1^ [105]	5.4 s
Ag10.6 nm	Tris-HOAc buffer in 10 M H_2_O_2_ 0.005 nM Ag^o^	0.122 s^−1^ [105]	582 s
TiO_2_ 1 - 24.4 nm Industrial	In aqueous NaCl solutions at temperatures of 25 and 37°C - pH ranging between 3.0 and 3.3.	3.3 × 10^−2^ h^−1^ [107]	21 hours

There are other studies in addition to those cited in Table 2 that have been conducted on dissolution of nanoparticles; unfortunately, many of them do not indicate dissolution rates, dissolution rate constants and the order of reaction. It is recognized that dissolution is a complex and dynamic process and that some nanoparticles are governed by complex dissolution kinetics. However, since biodurability is an important aspect in the risk assessment of nanoparticles, attempts should be made to determine their rate constants, and subsequently their half-times and lifetimes (biodurability).

## Conclusions

Dissolution, as a significant determinant of biodurability, has potential to influence the toxicity and pathogenicity of particles. Generally particles with lower biodurability have been shown to have lower pathogenicity and therefore dissolution studies may provide an indication to proceed from short-term toxicity to long-term chronic studies in a tiered-risk assessment strategy. Therefore, biodurability is an important parameter in the risk assessment of particles and fibres.

Dissolution of particles and fibres follow different reaction kinetics and therefore through the determination of dissolution rate constants, dissolution rates, rate constants, order of reaction (zero-, first- or second-order) it may be possible to assess their half-time and lifetime in different biological surroundings.

Many studies on dissolution of nanomaterials do not indicate dissolution rates, dissolution rate constants, or give the order of reaction. As much as dissolution is a complex process, it is recommended that studies on dissolution and biodurability of nanomaterials should include rate constants from which half-times and lifetimes can be derived. Attention should also be paid to carefully specify the parameters that affect the dissolution of particles and fibres including surface area, size, and type of media. Finally, as demonstrated in this review, dissolution studies of nanomaterials to assess biodurability should clearly state the conditions under which the dissolution studies were carried out with an important consideration that these studies are designed in a manner that the conditions mimic the relevant *in vivo* or environmental conditions.

## Abbreviations

nm: Nanometer
ZnO: Zinc oxide
CeO_2_: Cerium (II) oxide
Ag: Silver
*t*_*1/2*_: Half-time
*k*_*dis*_: Dissolution rate constant
τ: Lifetime
*k*_*SSA*_: Specific surface-area-normalised dissolution rate constant
CFT: Continuous-flow-through
μm: Micrometer
m: Meter
NaCl: Sodium chloride
Na^+^: Sodium ion
Cl^−^: Chloride ion
r: Rate
M: Mass
t: Time
dM: Change in mass
dt: Change in time
k: Rate constant
hr: Hour
L: Litre
A: Surface area
ng: Nanogram
g: Gram
s: Seconds
cm: Centimetre
D_0_: Initial fibre diameter
M: Initial mass
D: Fibre diameter
ρ: Density
G: Gibbs free energy
Si: Silicon
O: Oxygen
Ǻ: Angstrom
Ca^2+^: Calcium ion
H_2_O_2_: Hydrogen peroxide
HO_2_^−^: Hydroperoxyl
OCl^−^: Hypochloride
NOM: Natural organic matter
PVP: Polyvinylpyrrolidone
EDTA: Ethylenediaminetetraacetic acid
*k*_*phys*_: Physiological clearance
*k*_*decomp*_: Particle removal by breakage and disintegration
d: Diameter
Vm: Molar volume
